# Indigestion in the Horse*Read before the Massachusetts Veterinary Association.

**Published:** 1885-07

**Authors:** Charles J. Byrne


### Art. XVII.—INDIGESTION IN THE HORSE. *

BY CHARLES J. BYRNE, M. R. C. V. S.

In human medicine there are few subjects on which more has been written, and in veterinary less, than derangement of the digestive functions. Most of the veterinary authors have dealt with the subject in a trivial manner, therefore what I am about to read to you this evening is chiefly from notes of my own experience.

It is true, the horse is not subject to anything like the variety of gastric derangements that man is; although living in an artificial state, and on food, in a great measure, artificially prepared for his use, he is not allowed, like man, to overgorge his stomach and derange its functions by excesses in eating and drinking, smoking, etc., but, as a rule, has his food and water carefully dealt out to him in such quantity and quality as will generally insure him against dyspeptic attacks. Nevertheless, circumstances do occur, and these by no means rare, when the necessary attention is not paid to his diet, and serious derangement of his digestive apparatus is the immediate consequence.

Before dealing with indigestion, I shall endeavor to describe to you in as few words as possible, the process of digestion.

In the horse, as in other domestic animals, the digestive apparatus is constituted of a membraneous and contracted canal, which extends under the vertebral column from the mouth to the anus. This tube is lined by a mucous mem-

* Read before the Massachusetts Veterinary Association.

brane, the organization and functional properties of which, in its *abdominal* section, are of the greatest importance, being the principle seat of the essential phenomena of digestion; whilst the other parts, such as the *mouth*, *pharynx* and *œsophagus*, only serve to take, to prepare, and to introduce the alimentary materials.

Digestion may be said to be the process by which those parts of the food which may be employed in the formation and repair of the tissues, or in the production of heat, are made fit to be absorbed and added to the blood. Pressed by hunger and thirst, (sensations which express the necessity of repairing the general and continuous waste of the system) the animal secures the food which he meets with, or is given to him; previously, however, he takes care to subject it to an examination by sight and smell. Once in the mouth, the sense of taste must be satisfied; whatever is offensive is rejected, the residue is ground down by the teeth, and, while undergoing this process, becomes thoroughly impregnated with saliva, and is thus reduced to a soft, pulpy mass, which very much facilitates the next move, viz.: that of deglutition, or the passage of the food from the mouth to the pharynx into the æsophagus, from thence to the cardiac end of the stomach, where it is mixed with a quantity of mucous, which prepares it to be acted upon by the gastric juice of the true digestive, or pyloric end of that organ. By the agency of this fluid and the movement of the stomach, it is reduced to a semi-fluid consistency, and converted into a uniform pulp called chyme; this is then transmitted through the pylorics into the duodenum, and then mixed with the bile, and the pancreatic secretion and intestinal mucous. It is then in a fit state to have its nutritious parts taken up by the lacteal, which form a close system of vessels upon the mucous surface of the intestines and to be carried by them to the blood, while the indigestible or excrementitious part is moved onward by the peristaltic motion of the bowels and in due time expelled by the anus.

Having thus gone over in a brief manner, the healthy functions of the alimentary canal, I am brought to the more immediate subject of my paper, viz.: *Indigestion in the horse.*

In order that digestion may be perfect and easy, it is requisite that the food should be in a state of minute division. A weak stomach acts slowly or not at all, on tough masses of solid food; and horses, like men, have weak and dyspeptic stomachs. The greedy feeder swallows a great part of his food half masticated; the nibbler frequently disturbs his stomach and bowels with gas. In the former, the delayed masses undergo spontaneous changes which are prompted by the mere warmth and moisture of the stomach, gases are extricated, acids are formed, and the half-digested mass passes undissolved into the duodenum and becomes a source of irritation during the whole of its journey through the intestines. In the latter, (the nibbler) he disturbs his stomach and bowels so frequently with air, as to weaken the whole of the digestive powers, and to render him a dyspeptic subject. His rough coat, tight skin, and emaciated figure prove incontestably that his food passes through the alimentary canal without undergoing due conversion, or without his deriving that benefit from it which the healthy animal does.

I will endeavor to consider this subject under two heads, viz.: the mild form unattended by acute pain, called *chronic;* the other, which takes place suddenly, the symptoms of which are altogether more violent, and which is perilous to the existence of the animal, called *acute.*

In a state of chronic indigestion a horse does not thrive as others do, nor is he capable of doing the same amount of work; his appetite is fastidious—good, and even vivacious at one time, and only indifferent at another; it is sometimes depraved; he is fond of gnawing his rack and manger, is frequently found licking the walls, if any, or whitewash off the partitions. I have frequently seen horses eat any filth, and sometimes their own excrement. The skin, from sympathy with the alimentary canal, has an unhealthy appearance; the coat stares, and the animal becomes more or less hide-bound. The manure has not the natural appearance; at one time it is dark, and at another light colored, and has a very offensive smell. It is usually voided in small hard glazed balls, and if examined, will be found to consist of chopped hay and imperfectly chewed or champed oats. In the stable this is the usual state of the fæces, but in his weak state the horse is easily excited when at work, and purging is the consequence. The urine is also scanty and high colored.

The ordinary seat, particularly of chronic indigestion, is the mucous membrane of the stomach and intestines, and the disease may be defined to consist of a congested state of the blood vessels of that membrane; there is, consequently, a want of the proper secretions and constipation is the result. This torpid, or abnormal state may be produced by many causes, such as irregularity in the quality and quantity of the food, imperfect mastication of food in consequence of diseased or irregular teeth, or from greedy or ravenous feeding, long fasting from food and water, cribbing and quiding from irregularity in the teeth, or bots, previous attacks of acute indigestion, or irregular exercise, disease of the liver, &c. These are among the principle causes of indigestion in the horse.

There are few animals, in their natural state, that are supposed to spend more of their time in feeding than the horse does; and the fact that he has no biliary receptacle, proves the necessity of his doing so, and ought to teach a lesson to all those who are interested in his well-being, to copy the dictates of nature by feeding him frequently.

A long fast renders a horse voracious, like the natural greedy feeder; his food is bolted without sustaining that thorough grinding with the teeth so essential to a healthy system. If allowed, he will sometimes overgorge himself with an indigestible mass to such an extent as to bring about the partial or entire suspension of the movement and secreting power of the stomach, and thus put his life in serious danger from fermentation and rupture.

Wheat or green food is most likely to produce this effect, and especially so, if he be put to severe work immediately, or very soon after feeding. New oats or new hay will also produce it; also debility of the digestive organs, or an unhealthy state of the general system; or it may be caused by excessive fatigue, producing general weakness; cold water given in large quantities too soon after eating, by washing the ingesta from the stomach before it is properly chymified, will cause it; allowing the body to cool too quickly when warm; bad treatment; and so will irregularity of work or nervous excitement of any kind.

The symptoms of the acute form of this disease will depend altogether upon its particular seat. If the horse has overloaded his stomach, he will have excessive nausea, expressed by the drooping of the head, turning up of the nose, attempts to vomit, eructations, a slow weak pulse and great prostration and heaviness, distention of the abdomen and colicy pains. *There may* or may not be sympathetic affections of the *brain*, producing stupor and staggers.

There are many cases where this symptom is not present, though the stomach is distended to the utmost. The horse is frequently attacked in the middle of his work, becomes uneasy and will lie down, and at times at full length, for a considerable period; the extremeties are cold, the mucous membranes are not much injected, the bowels are constipated, the mouth dry and clammy, and the horse has a peculiar haggard countenance, which becomes more and more ghastly as the disease advances; should the stomach burst he becomes pulseless, cold sweats break over him, the mucous membranes are pale as in death, he makes frequent attempts to urinate and to force everything from the rectum, he reels, staggers, and very soon falls headlong into a corner of the building and dies.

Although cases of acute indigestion, such as I have just described, where the disease is altogether confined to the stomach, are by no means rare, still the true seat of the great bulk of our acute cases will be found in the cœcum and colon, that mighty receptacle which indeed, might be called the horse's second stomach, for it is indisputable that large masses of nearly indigestible matter lodge there, no doubt for the wise purpose of extracting from it the last particle of its nutritive properties; and it is natural to expect that where there is the greatest accumulation of aliment, there should be the most frequent derangement of function, and so we find it, for in large practice the treatment of this form of indigestion becomes almost a daily occurrence.

The large masses so frequently accumulated in this part of the bowels, in the heavier classes of horses particularly, becomes a frequent source of irritation, producing enteritis; but far more frequently, that form of indigestion so well known to every amateur in veterinary science under the designation of flatulent colic. The causes of this form of indigestion are nearly the same as those which produce it in the stomach, and which I have enumerated before; but the most common are eating green food to excess, and if wet, the danger is greater.

All kinds of new corn and hay have a great tendency to fermentation, or large masses of any indigestible matter, which has been imperfectly chymified from a defective state of the stomach.

If the horse be attacked in the stable, he ceases to eat, and seems to feel a disgust for all species of food and drink ; the head is lowered and occasionally thrown from side to side ; if he is at work he becomes suddenly heavy and idle, or perhaps works with more precipitation than usual ; he stops, scrapes with his feet and makes some contortions, and strives to lie down ; consents to continue his journey only when excited by the voice of his driver and whip ; he does not go far before he stops again, looks at his belly and groans, strikes at it with his hind feet, lowers the head and neck, and makes another effort to lie down in spite of whip and voice.

His driver will find it necessary to get him into some stable with as little delay as possible, for very soon the disease becomes aggravated ; the paroxysms of pain become more frequent and violent ; the horse is out of breath and covered with sweat ; he lies down and rolls from side to side ; the pulse as yet not much disturbed, except during the paraxysms ; the belly is swollen ; the nostrils are distended, and the whole frame seems to quiver with agitation ; later on he sustains himself with more difficulty, spreads his legs to support himself upright, and will often be found to lean against the stable or wall for support. He now lies down with more caution ; he dreads the danger of doing so ; he often stretches himself and makes vain attempts to pass his manure and urine ; feels a desire to vomit, manifested by the elongation of the head and neck ; he frequently belches up gases, which is sometimes accompanied with liquids, mixed with particles of food, which escape by the nose and mouth. The swelling of the belly now augments with fearful rapidity ; the right flank becomes elevated ; the countenance is expressive of the most intense suffering ; the pulse is nearly imperceptible ; he is now nearly insensible to everything that is around him, and to everything that you do to him, except, perhaps, that he will refuse to take any draught with all the energy with which he is still capable. The anus, forced out by the intestines, forms a sort of soft tumor which elevates the tail. The skin is now covered with cold sweat, and the pulse completely gone ; the air can scarcely penetrate the respiratory organs; the blood circulates with difficulty in the vessels; asphyxia becomes imminent; the animal staggers and may fall heavily; sometimes he is relieved by sitting on his haunches like a dog, but this relief is deceptive; it is the result of rupture of the stomach, intestines, or diaphragm allowing displacement of gas. A moment after he becomes comatose; their is no further relief for him, his vital energy is exhausted and he dies.

I now come to the treatment of the various forms of digestive derangements which I have endeavored to describe in this paper, and in doing so it will be necessary that we consider carefully the causes by which they are produced. In all cases, but particularly in those of the milder form of indigestion, much may be learned from the groom or stable attendant, and it is of the utmost importance to the success of our treatment, that as much of the history of the case be got from him as possible.

*First.*—If the horse has been irregularly fed, or his work more than ordinarily severe, he should have absolute rest, change of diet, a slight dose of physic, and a few vegetable tonics.

*Second.*—If the cause is greedy feeding, have his corn ground, hay cut, and a muzzle put on at night to prevent him filling his belly with idigestible matter.

*Third.*—If the horse is a cribber, let him wear a strap round his neck, and feed totally off the ground.

*Fourth.*—If the teeth are the cause, remove decayed ones, and the protruding parts of others cut off and rash down.

*Fifth.*—If from *bots* or other parasites, prescribe for their removal and the disease will subside.

Indeed, many of the milder cases of indigestion may be cured by rest, change of diet and stable management; the body and legs kept warm with clothing and bandages; the stall or box well fitted and kept clean; the food given often and in small quantities—in *Summer* a vernal grass or green food—in *Winter* carrots and half shorts or bran, with his corn or oats. Occasionally small doses of physic followed with tonics. If the animal bites the walls or licks the lime of the same, or eats earth, it is indicative of heartburn. Discontinue his oats and corn for a few days and feed on boiled oats and bran mashes and bi-carb. soda in half to one ounce doses three times a day, and also, as in other cases, small doses of physic and tonics. Severe purging does harm, but mild laxatives with good nursing, change of diet given in small quantities, with moderate slow exercise, will often be all that is necessary.

Now, if the case be an acute one, and the stomach overloaded, it becomes a very serious matter, and it will depend in a great measure on the extent to which that organ is packed, whether any treatment be of the slightest use.

I have examined horses that have died of rupture of the stomach, and found upwards of forty pounds of food in that organ; the ingesta was nearly dry, and had no appearance of having been acted upon in the slightest degree by the gastric juice; that secretion must have been entirely suspended, as well as the movement of the stomach, by the excessive weight of its contents. Medical treatment in cases of this kind must, I fear, ever prove futile in the horse; but fortunately, they are not all so bad, and plenty of cases will be met with where the stomach is overloaded, but only to such a degree that active treatment is often efficacious. In such cases, the object to be aimed at is the expulsion of the contents of the stomach, and the most natural way is to rouse that organ to increased action; and to accomplish this I know of nothing better than stimulants and purgatives. Apply mustard, hot fermentations, warm injections, and order as much gruel to be given as he will take.

While the walls of the stomach are so distended, there is no danger of inflammation; should there be any cerebral symptoms, such as heaviness of the head, leaning the head on the manger, or thrusting it against the wall, a good free bleeding should be added to the stimulants and purgatives.

If the disease should take the tympanitic or gaseous form, the cœcum or colon is its usual seat; the cures for it are innumerable; every quack has his own infallible specific, and most veterinarians have a remedy which they think nearly a cure; but although many cases, no doubt, are cured, still it is beyond doubt many die, and from gaseous distention alone, without a particle of inflammation. Like the previous cases I have dealt with, where the stomach is distended with food, expulsion is the object we have to attain. To have the bowels distended with gas, we must have fermentation; and to have fermentation there must be a mass of imperfectly digested matter in the gut. All agree giving the most powerful stimulants, and there are few who don't think it necessary to combine with them some active purgative.

I condemn the fashion of walking and trotting the animal in such cases.

In despite of all treatment, however sound, both in theory and practice, our cases will sometimes die, and others appear upon the point of bursting.

In such cases I have used the trocar in thirty-one instances, with nine successful results. I believe, however, if it were used earlier than it usually is, the results would be more successful.

